# Chitooligosaccharide enhanced the efficacy of *Bacillus amyloliquefaciens* CAS02 for the control of tobacco black shank

**DOI:** 10.3389/fmicb.2023.1296916

**Published:** 2023-11-24

**Authors:** Xiangnan Zeng, Xin Zhang, Bo Peng, Bingyue Xie, Yuan Yuan, Hui Yao, Xiangwei You, Jianyu Wei, Yiqiang Li

**Affiliations:** ^1^China Tobacco Guangxi Industrial Co., Ltd., Nanning, China; ^2^Marine Agriculture Research Center, Tobacco Research Institute, Chinese Academy of Agricultural Sciences, Qingdao, China

**Keywords:** biocontrol bacteria, chitooligosaccharide, plant defense signal marker gene, rhizosphere soil microorganism, tobacco black shank

## Abstract

**Introduction:**

Tobacco black shank is a devastating soil-borne disease caused by the oomycete *Phytophthora nicotianae*, severely hamper tobacco production worldwide. However, the synergistic effect of biocontrol bacteria and marine polysaccharides/oligosaccharides on tobacco black shank control was few documented.

**Methods:**

In this study, *Bacillus amyloliquefaciens* CAS02 (CAS02) and chitooligosaccharide (COS) were screened firstly, and their synergistic antagonistic effect against *P. nicotianae* and the underlying mechanism were investigated *in vitro* and *in vivo*.

**Results:**

*In vitro* experiments showed that, compared with the application of CAS02 or COS alone, co-application of CAS02 and COS significantly increased the inhibition rate against *P. nicotianae* by 11.67% and 63.31%, respectively. Furthermore, co-application of CAS02 and COS disrupted the structure of mycelia to a greater extent. The co-application of CAS02 and COS showed synergistic effect, with the relative control effect maintained above 60% during the 60-day pot experiment, significantly higher than that of application CAS02 or COS alone. The combined application of CAS02 and COS reduced the relative abundance of *P. nicotianae* in the rhizosphere soil and increased the relative abundance of bacterial taxa potentially involved in disease suppression, such as *Nocardioides*, *Devosia* and *Bradyrhizobium*. Meanwhile, CAS02 and COS synergistically activated salicylic acid (*SA*), ethylene (*ET*), and hypersensitive response (*HR*) defense signaling pathways in tobacco plants.

**Discussion:**

Our findings demonstrate that co-application of CAS02 and COS remarkably improve the relative control effect against tobacco black shank through multiple pathways and provide a promising strategy for the efficient green control of tobacco black shank.

## 1 Introduction

Tobacco black shank is a devastating soil-borne disease caused by the oomycete *Phytophthora nicotianae* that causes severe stem and root rot (Guo et al., 2020). Because the pathogen can survive in soil and plant tissues at all stages of growth, the black shank is one of the most difficult diseases to control (Liu et al., 2020). Within a few decades of its discovery, the disease had spread to tobacco-growing areas worldwide and cause great economy loss (Gallup et al., 2018; Ma et al., 2018). The traditional approaches to control tobacco black shank include the application of fungicides (Han et al., 2016), crop rotation (Niu et al., 2017) and selection of disease-resistant varieties (Deb et al., 2018), which have many limitations. For example, the application of pesticide not only cause resistance but also inevitably result in pesticide residues, which threats human health (Stumbriene et al., 2018). Therefore, it is urgent to explore green, efficient and sustainable methods to control tobacco black shank.

Biocontrol bacteria provide a promising approach for controlling tobacco soil-borne diseases, which act through antagonism, lysis, competition, promotion and micro ecological regulation to achieve disease control (Tao et al., 2020; Dey and Ghosh, 2022). The effectiveness of *Bacillus subtilis* and *Pseudomonas chlororaphis* for plant disease control has been widely reported (Han et al., 2016; Das, 2017; Rajaofera et al., 2018). However, despite the promising application of environment friendly biocontrol, applying a single biocontrol bacterium has many problems compared to chemical pesticides, such as narrow spectrum of target pathogens, susceptibility to environmental factors, and poor control effect in practical applications (Fira et al., 2018). Therefore, the methods to improve efficacy of biocontrol bacteria have attracted increasing attentions of researchers. Marine polysaccharides/oligosaccharides are natural biological compounds with biodegradability, biocompatibility, and bioactivity properties (Chen et al., 2021; Yermak et al., 2022). Previous studies have reported that marine polysaccharides/oligosaccharides with antimicrobial activity can be fully synergized with biocontrol bacteria to improve the biocontrol effect of biocontrol bacteria effectively (Das et al., 2015; Hu et al., 2016; Muxika et al., 2017). For example, Lin et al. (2020) found that biocontrol strain J02 showed more excellent disease suppression when applied synergistically with 1% chitosan, effectively suppressing soft rot disease on radish roots and reducing the severity of cabbage black spot by about 70% or more. However, the synergistic effects of marine polysaccharides/oligosaccharides and biocontrol bacteria on controlling tobacco black shank and the underling mechanism are poorly documented.

Therefore, in this study, we first screened *Bacillus amyloliquefaciens* strain CAS02 and chitooligosaccharide (COS), which have a strong ability to inhibit *P. nicotianae* as potential biocontrol strain and biocontrol oligosaccharide, respectively. Following observation of the effect of individual and co-application of CAS02 and COS on *P. nicotianae* morphology *in vitro*, we explored the control efficiency against tobacco black shank using pot experiments and analyzed the effect on the rhizosphere soil microbial community using high-throughput sequencing. Moreover, the expression level of resistance related genes in tobacco plants with individual or co-application of CAS02 and COS was also investigated. The findings of this study will help to develop a new *P. nicotianae* inhibitors based on the combination of oligosaccharides and biocontrol bacteria for the efficient green control of tobacco black shank.

## 2 Materials and methods

### 2.1 Experiment materials

*Phytophthora nicotianae* was isolated from infected tobacco plants, supplied by the Key Laboratory of Tobacco. The tested biocontrol bacteria (*Bacillus amyloliquefaciens* CAS02, *Bacillus aureus* 3 T33, *Bacillus megaterium* BB22, *Bacillus adenii* BBS23, *Bacillus natto* NATT0, *Bacillus alpine* 7, *Bacillus amyloliquefaciens* SB-1 and *Bacillus amyloliquefaciens* SB-2) were provided by the Institute of Tobacco Research, Chinese Academy of Agricultural Sciences and their isolated sources were list in Supplementary Table S1. Some of these bacterial species were proven antagonistic activity against bacterial, fungal and oomycete pathogens, such as *Ralstonia solanacearum*, *Alternaria alternate*, *Mycosphaerella graminicola* (Kildea et al., 2008; Chu et al., 2022; Yuan et al., 2023). Chitooligosaccharide (COS) was purchased from Qingdao Songtian Biotechnology Co., Ltd. Alginate oligosaccharide (AOS) and carrageenan oligosaccharide (CAOS) were purchased from Qingdao Bozhihuili Biotechnology Co., Ltd. *Enteromorpha prolifera* polysaccharide (EP) and fucoidan oligosaccharide (FOS) were purchased from Qingdao Haid Biological Group Co., Ltd. and Henan Zhongchen Biotechnology Co., Ltd. respectively. Tobacco variety Small Gold 1025 is susceptible to black shank disease. The culture media used include nutrient agar (NA), nutrient broth (NB) and oats (OA) medium.

### 2.2 Inhibitory effect of biocontrol bacteria and polysaccharides/oligosaccharides on *Phytophthora nicotianae*

The bacterial strains and marine polysaccharides/oligosaccharides with inhibitory activity against *P. nicotianae* were screened by the dual culture and mycelial growth relative inhibition methods according to Anith et al. (2021) and Wang et al. (2018), respectively. Four 5-μL drops of different biocontrol bacteria solutions (OD_600_ value of 0.4) were placed around the OA plate (20 mm from the center of the OA plate), and sterile water was added dropwise as the control. Different marine polysaccharides/oligosaccharides were added to the OA medium with a uniform final concentration of 5 mg/mL. OA medium without marine polysaccharides/oligosaccharides was used as the control. After the mycelia fully covered the OA plate, mycelia disks of *P. nicotianae* (5 mm dimeter) were obtained and placed in the center of OA plates. Each treatment was replicated thrice and all plates were incubated at 28°C for 4 days. The colony diameter was determined using the vertical cross method, and the mycelial growth relative inhibition rate was calculated using the following equation.

Mycelial growth relative inhibition rate (%) = ([(control colony diameter − 0.5) − (treated colony diameter − 0.5)]/(control colony diameter − 0.5)) × 100%.

Different masses of COS were added to OA medium to obtain different final concentrations (0 mg/mL, 0.075 mg/mL, 0.15 mg/mL, 0.3 mg/mL, 0.6 mg/mL, and 1.2 mg/mL). Mycelial relative inhibition rates were calculated as mentioned above. The EC_50_ values for COS inhibition of *P. nicotianae* were calculated (Wang et al., 2020).

### 2.3 Synergistic application of CAS02 and COS *in vitro*

To determine whether COS addition changed the morphology of the biocontrol bacteria CAS02, subsequently affected its growth and biocontrol ability, growth curve assay and scanning electron microscopy (SEM) were performed (Han et al., 2016; Zhang et al., 2022). Strain CAS02 was first activated in NA culture medium and then scaled-up NB medium for cultivation. After cultivation, the 20 μL bacterial suspension was inoculated into conical flasks containing 20 mL NB medium with 3.76 mg COS (188 μg/mL, COS treatment) or without COS (CK) added and then incubated at 28°C and 180 rpm. Each treatment was repeated thrice. The OD_600_ value of the culture liquid was measured every 3–4 h during the shaking cultivation. After 16 h of shaking cultivation, the culture liquid from both the control and experimental groups was centrifuged at 4°C and 6,000 rpm for 5 min to collect bacterial cells. After centrifugation, the bacterial samples in control and COS treatment were fixed with 2.5% glutaraldehyde aqueous solution for 4 h at 4°C and then washed six times with phosphate buffered saline (PBS) solution for 30 min each time. After fixation, the cubes were dehydrated with different concentrations of ethanol (30, 50, 70, 90, and 100%) for 30 min. The samples were added to isoamyl acetate followed by CO_2_-mediated critical point drying. Subsequently, samples were gold plated, and electron micrographs were obtained using a JSM-840 scanning electron microscope (JEOL, Tokyo, Japan).

The antagonistic activity of individual and co-application of CAS02 and COS against *P. nicotianae in vitro* was determined using a plate assay (Guo et al., 2020). Four treatments were set up: (1) control: blank OA without CAS02 or COS added; (2) COS: COS was added at the concentration of 188 μg/mL; (3) CAS02: CAS02 strain solution (OD_600_ value of 0.4) was added; (4) CAS02-COS: 188 μg/mL COS and CAS02 strain (OD_600_ value of 0.4) were added. The mycelial growth status was observed and the inhibition rate of mycelial growth was calculated as described above.

Furthermore, the effect of individual and co-application of CAS02 and COS on the mycelial morphology and ultrastructure of *P. nicotianae* was investigated using SEM and transmission electron microscopy (TEM) analysis. In brief, the mycelia of *P. nicotianae* at the edge of inhibition zone in different treatments were cut into 0.5 × 0.5 × 0.5 cm^3^ cubes. The samples were processed as described above (Zhang et al., 2022) and were observed using SEM. For TEM analysis, samples were dehydrated and embedded and ultrathin sections were obtained using an ultramicrotome (Wang et al., 2022). Mycelial structural changes were observed by double staining with uranyl acetate and lead nitrate using a JEM-1200EX electron microscope (JEOL Ltd).

### 2.4 Synergistic application of CAS02 and COS *in vivo*

The effects of individual and co-application of COS and CAS02 to control tobacco black shank were investigated by pot experiments. The tested soil is field soil (loamy type), uninfected with *P. nicotianae*, collected at the Jimo base filed of the Institute of Tobacco Research, Chinese Academy of Agricultural Sciences. This filed has been cultivated with wheat and maize in recent years. To prepare disease soil, the millet with *P. nicotianae* was mixed evenly with this healthy filed soil, in a mass ratio of 1:100. Tobacco seedlings with basically uniform growth containing 5 leaves were transplanted to pots containing 200 g of potting soil and 2 g millet with *P. nicotianae* (1 plant per pot, top diameter 12 cm × 9.5 cm × 10 cm). Four treatments were set up as follows: (1) Control: no CAS02 suspension or COS added; (2) CAS02: 25 mL CAS02 suspension (OD600 value of 0.4) was added; (3) COS: 36 mg COS was added; (4) CAS02-COS: 36 mg COS and 25 mL CAS02 suspension (OD_600_ value of 0.4) were added. Each tobacco plant was inoculated via irrigation with 25 mL CAS02 suspension, and the 36 mg oligosaccharide was uniformly mixed into potted soil. Each treatment consisted of three replicates, each including 15 seedlings. The seedlings were then incubated in a climatic chamber (28°C, 70% relative humidity; light: dark cycle, 12:12 h).

The disease incidence with individual or co-application of CAS02 and COS was recorded on the days 10 and 25 after pathogen inoculation, and the disease index (DI) and relative control effect were calculated as follows:

DI = [(a × 0) + (b × 1) + (c × 3) + (d × 5) + (e × 7) + (f × 9)]/[(a + b + c + d + e + f) × 9] × 100.

Where a, b, c, d, e, and f are the number of plants in each disease category (Zhang et al., 2017).

Relative control effect (%) = [(Disease index of control − Disease index of treatment)/Disease index of control] × 100.

Tobacco leaf samples were collected from the same part of each plant on the day 10 after pathogen inoculation, then washed with cold distilled water, frozen with liquid nitrogen, and stored at −80°C for further determination of defense signaling marker genes of tobacco plants (Tahir et al., 2017). On the day 25, the plant height and maximum leaf area were measured. Soils tightly adhering to tobacco roots were also sampled as rhizosphere soils using hand-shaking method (Yin et al., 2022; Jun et al., 2023). In briefly, the soil in the root zone was excavated, and soil that was not tightly bound to the roots was gently removed by shaking. The soil samples adhering to the roots were carefully collected and defined as rhizosphere soil. All soil samples were immediately stored at −80°C for further soil microbial community analysis.

### 2.5 DNA extraction, quantitative analysis of *Phytophthora nicotianae*, PCR amplification and high-throughput sequencing of rhizosphere soil microorganism

Total genomic DNA was extracted from 0.5 g rhizosphere soil using the FastDNA Spin Kit (MP Biomedicals, CA, United States) according to the manufacturer’s instructions. The quality and concentration of DNA was evaluated using a NanoDrop 2000 spectrophotometer (NanoDrop, ND2000, Thermo Scientific, DE, United States). The abundance of *P. nicotianae* in the rhizosphere soil was determined by quantitative real-time PCR (qPCR) using primers SP (5′-TGAAGAACGCTGCGAACTGC-3′) and AP (5′-CTGACATCTCCTCCACCGACTA-3′) (Wang et al., 2020). qPCR reactions were carried out in 20 μL reaction volume containing 2.0 μL of DNA, 10.0 μL of SYBR premix (TAKARA), 0.4 μL of ROX Reference Dye II (50×), 0.4 μL of SP (10 μM/μL), 0.4 μL of AP (10 μM/μL), and 6.8 μL ddH_2_O. The PCR condition was set at 94°C for 5 min; 40 cycles of 94°C for 20 s, 65°C for 40 s, and 72°C for 40 s.

The V3-V4 region of the 16S rRNA gene were amplified using primers 338F and 806R. The 25 μL reaction solution consisted of 10 ng DNA, 0.2 μm each primer, and 15 μL Phusion® High-Fidelity PCR Master Mix (New England Biolabs). PCR was carried out in triplicate at 98°C for 2 min, followed by 25 cycles of 98°C for 15 s, 55°C for 30 s, 72°C for 30 s, and finally prolongation at 72°C for 5 min. After purification, detection, and quantification of PCR products, the library was constructed and sequenced at an Illumina NovaSeq PE250 platform. Raw sequences were quality-filtered and the processed high-quality sequences were clustered into operational taxonomic units (OTUs) at a 97% sequence similarity level using Usearch. A representative sequence (the most abundant) of each OTU was selected for searching against the SILVA database (version 132) with a confidence cut-off value of 0.7. To eliminate the effects of different sequence numbers among the samples on the bacterial community identified, the number of sequences per sample was rarefied to the smallest sample size.

### 2.6 Determination of relative expression level of defense signal marker genes in tobacco plants

To assess the systemic defenses induced by the co-application of CAS02 and COS, the expression level of defense signaling marker genes in tobacco leaves were evaluated. Total RNA was extracted from 0.1 g tobacco leaves on the day 10 after inoculation with the *P. nicotianae* using the Steady Pur plant RNA extraction kit (AG21019, Accurate Biotechnology (Hunan) Co., Ltd., China), following the manufacturer’s instructions. The extracted total RNA was separated and purified by agarose gel electrophoresis. The first-strand cDNA was synthesized using RT Mix Kit with gDNA Clean for qPCR (AG11728, Accurate Biotechnology (Hunan) Co., Ltd., China). The corresponding primers of the defense signaling marker genes were designed as previously described (Tahir et al., 2017; Zhao L. et al., 2019; Li et al., 2020) and were listed in Supplementary Table S2.

### 2.7 Statistical analysis

The mean values of all data were derived from three biological replicates, and the standard errors of the means were calculated. One-way analysis of variance (ANOVA) was performed followed by Duncan’s test to compare significant differences among different treatments at *p* < 0.05 using SPSS software (20.0, IBM, United States). In high-throughput analysis, the principal co-ordinates analysis (PCoA) was conducted based on Bray-Curtis method to evaluate the effect of individual and co-application of COS and CAS02 on rhizosphere soil bacterial community composition using vegan package in R version 3.6.3. To examine the correlation between the relative abundance of rhizosphere soil microbial taxa at genus level and disease indices, spearman’s correlation analysis was carried out using agricolae package in R version 3.6.3. Furthermore, to further investigate the response of bacterial taxa to the individual or combined application of CAS02 and COS, two group comparisons were conducted at genus level using student’s t-test at *p* < 0.05.

## 3 Results

### 3.1 Inhibitory effect of biocontrol bacteria and marine polysaccharides/oligosaccharides on *Phytophthora nicotianae*

**Figure 1:**
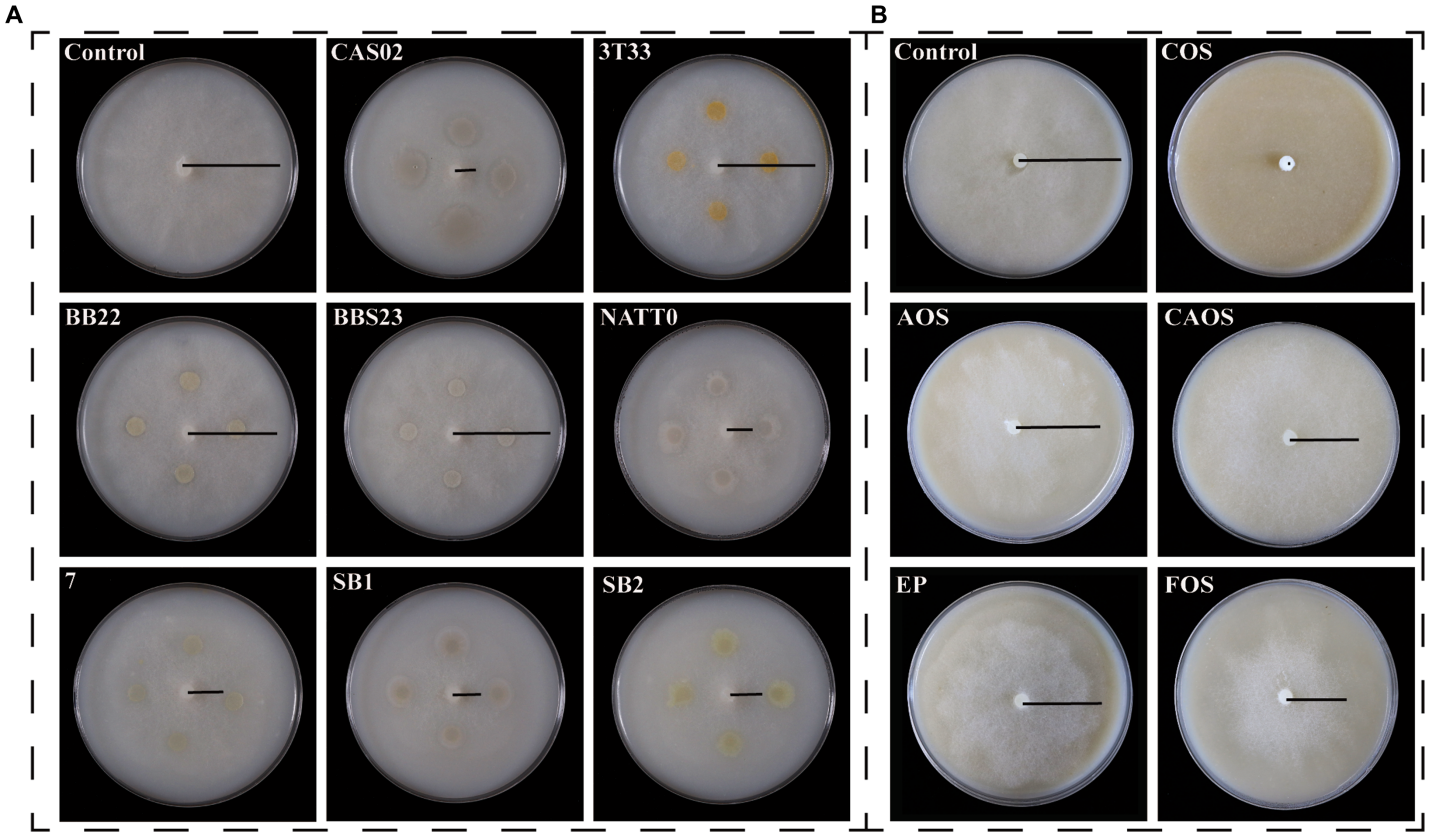
Inhibitory effects of biocontrol bacteria **(A)** and marine polysaccharides/oligosaccharide **(B)** on *Phytophthora nicotianae*. CAS02, *Bacillus amyloliquefaciens* CAS02; 3 T33, *Bacillus aureus* 3 T33; BB22, *Bacillus megaterium* BB22; BBS23, *Bacillus adenii* BBS23, NATT0; *Bacillus natto* NATT0; 7, *Bacillus alpine* 7; SB1, *Bacillus amyloliquefaciens* SB1; SB2, *Bacillus amyloliquefaciens* SB2; COS, chitooligosaccharide; AOS, alginate oligosaccharides; CAOS, carrageenan oligosaccharide; EP, enteromorpha polysaccharide; FOS, fucoidan oligosaccharide.

Among the eight selected biocontrol strains, strain CAS02 had the highest inhibition activity against *P. nicotianae*, which inhibited the growth of the entire colony obviously, resulting in a prominent circular inhibition zone (Figure 1; Supplementary Table S3). Furthermore, COS had the strongest inhibitory ability against *P. nicotianae* among the selected marine polysaccharides/oligosaccharide (Supplementary Table S4). When treated with 5 mg/mL COS, the mycelial growth of *P. nicotianae* stopped, and the colony diameter decreased by 100% (Figure 1; Supplementary Table S4). As shown in Supplementary Figure S1, the relative inhibition rate of COS increased with the increase in COS concentration. The toxicity regression equation was calculated as y = 1.2401x + 2.179 (*r*^2^ = 0.9955; where x is the logarithmic dose and y is the probability of death value), and its EC_50_ value was 188 μg/mL.

### 3.2 Effects of co-application of CAS02 and COS on the inhibition rate against *Phytophthora nicotianae in vitro*

**Figure 2:**
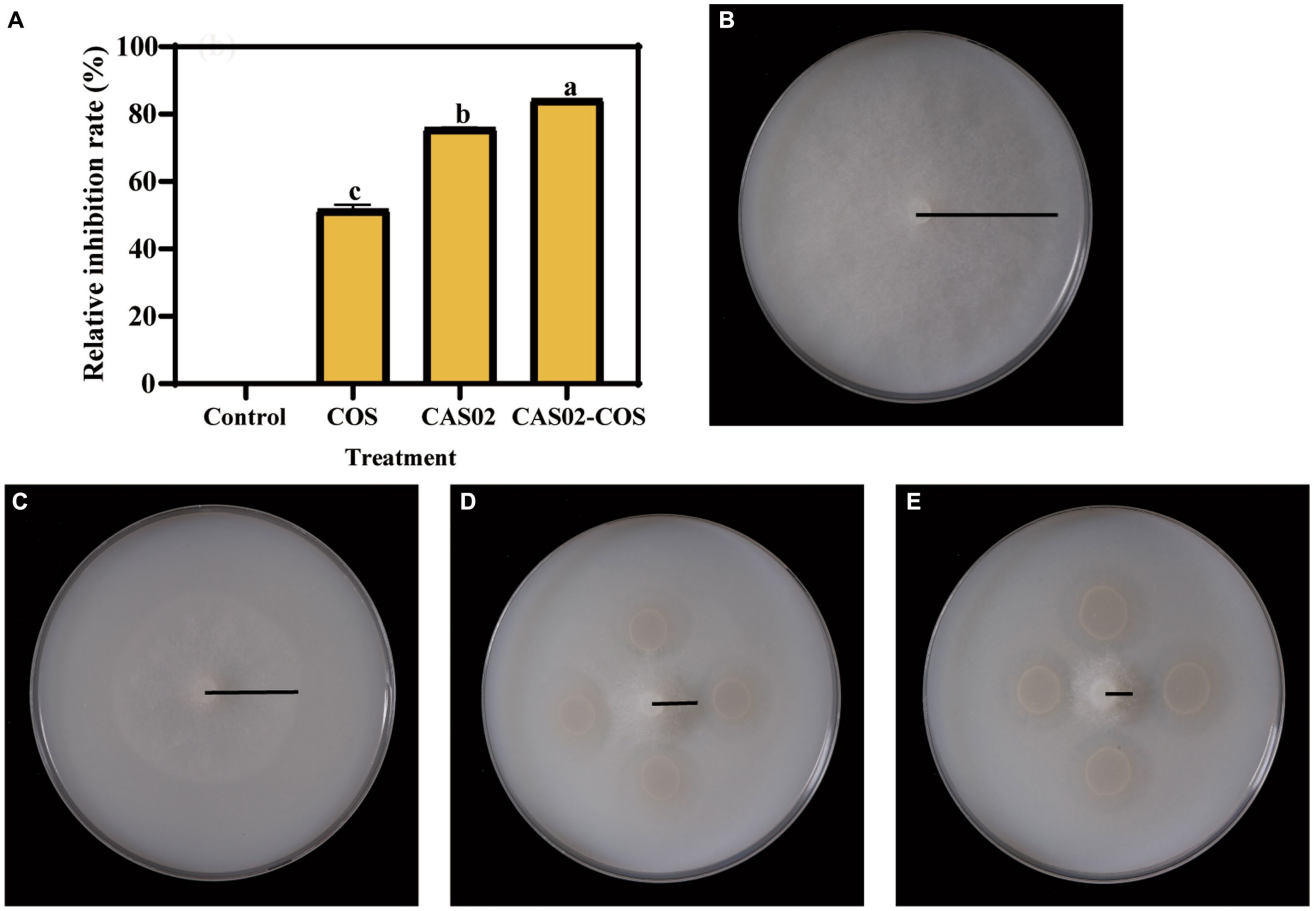
Synergistic inhibitory activity of CAS02 and COS against *P. nicotianae*. **(A)** The relative inhibition rate of CAS02 and COS in the synergistic inhibition of *P. nicotianae*. Growth of *P. nicotianae* mycelium in control **(B)** and after application COS alone **(C)**, CAS02 alone **(D)** and co-application of CAS02 and COS **(E)**.

The effect of COS on the growth ability of strain CAS02 was investigated by measuring the growth curve of CAS02. At a concentration of 188 μg/mL, COS did not have a negative impact on the logarithmic growth phase of strain CAS02. Instead, it extended the logarithmic growth time and increased the bacterial quantity of strain CAS02 in the stable growth phase (Supplementary Figure S2A). SEM was used to investigate whether COS addition at a concentration of 188 μg/mL changed the morphology of strain CAS02 or not. The morphology of strain CAS02 treated with COS did not differ from that without COS (Supplementary Figures S2B,C). The *in vitro* inhibition effect of CAS02 and COS synergistic application was determined by plate test. As shown in Figure 2, compared with application CAS02 or COS alone, co-application of CAS02 and COS significantly increased the inhibition rate against *P. nicotianae* by 11.67% and 63.31%, respectively.

### 3.3 Effects of co-application of CAS02 and COS on the morphology of *Phytophthora nicotianae*

**Figure 3:**
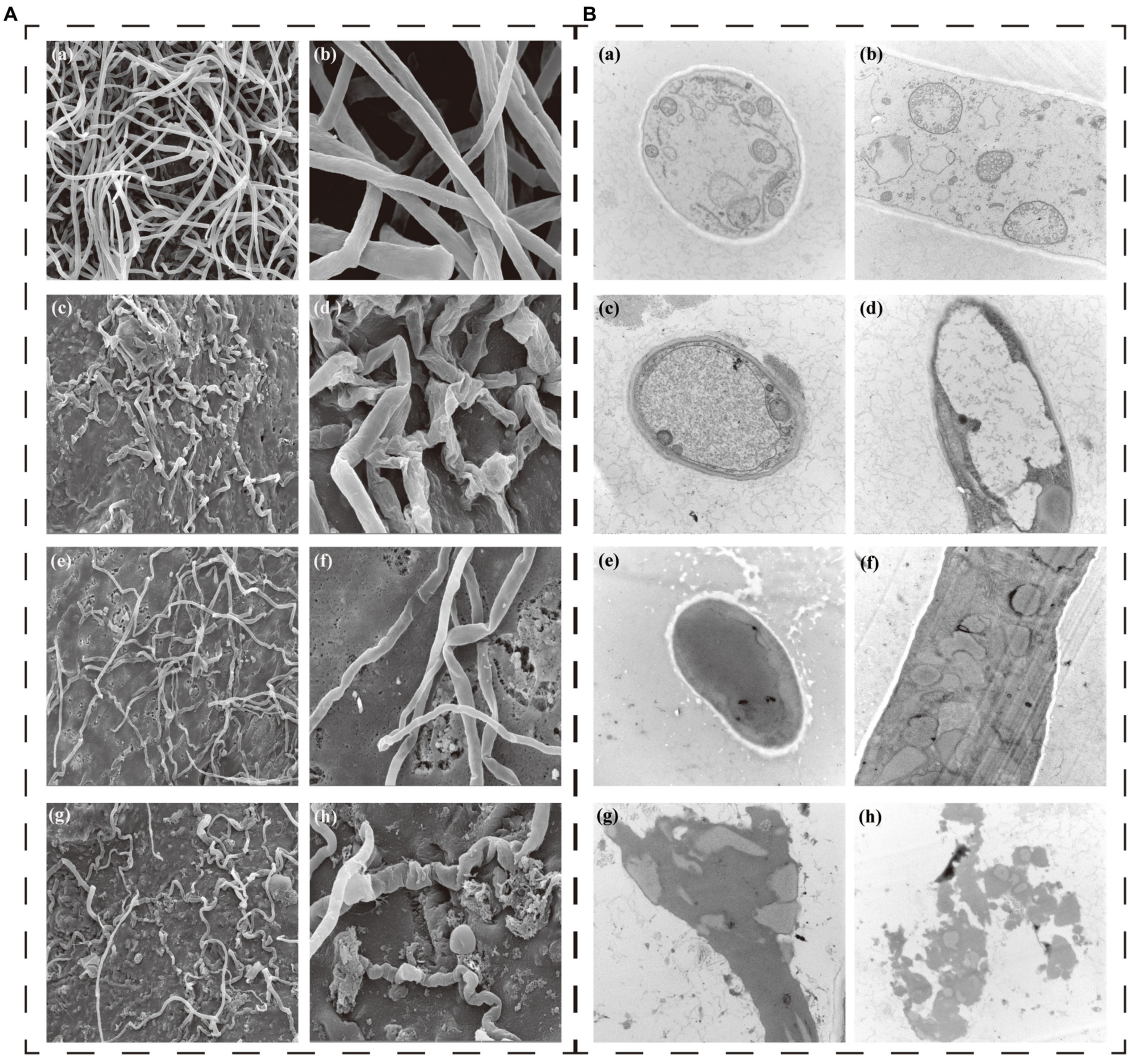
Effect of CAS02 and COS on the morphology **(A)** and ultrastructure **(B)** of the pathogen *P. nicotianae*. In both **A** and **B** (a,b) show healthy mycelia in control. (c,d) Present the effects of application CAS02 alone. (e,f) Present the effect of application COS alone. (g,h) Present the effect of synergistic application of CAS02 and COS.

SEM showed that the mycelia of *P. nicotianae* without CAS02 or COS treated had a complete surface, uniform thickness, smooth lines, good extension, and intact tubular structure (Figures 3Aa,b). However, after co-cultured with CAS02, the mycelia of *P. nicotianae* was substantially twisted, collapsed, and deformed (Figures 3Ac,d). The mycelia with COS application were thinned, substantially folded locally, and tended to break obviously (Figures 3Ae,f). The external structure of mycelia with co-application of CAS02 and COS was severely damaged, and the mycelium was obviously thinned, depressed, twisted to a greater extent, even the whole mycelium was dried and broken (Figures 3Ag,h).

TEM analysis showed that the control mycelium had an intact cell structure with uniformly thin and thick cell walls, uniform distribution of organelles in the cytoplasm, and uniformly dense protoplasm *in vivo* (Figures 3Ba,b). However, compared with the control treatment, CAS02 caused the obvious blurring and thinning of the mycelial cell wall of *P. nicotianae* (Figures 3Bc,d). COS resulted in the mycelial cell wall of *P. nicotianae* tending to be blurred, and the organelle structure was also blurred (Figures 3Be,f). The co-application of CAS02 and COS led to severe disruption of the mycelial structure of *P. nicotianae*. The cell wall and cell membrane were dissolved, the cytoplasm was mixed, the organelle structure was blurred, and the cell contents were severely lost (Figures 3Bg,h).

### 3.4 Effects of co-application of CAS02 and COS on tobacco black shank under greenhouse conditions

**Figure 4:**
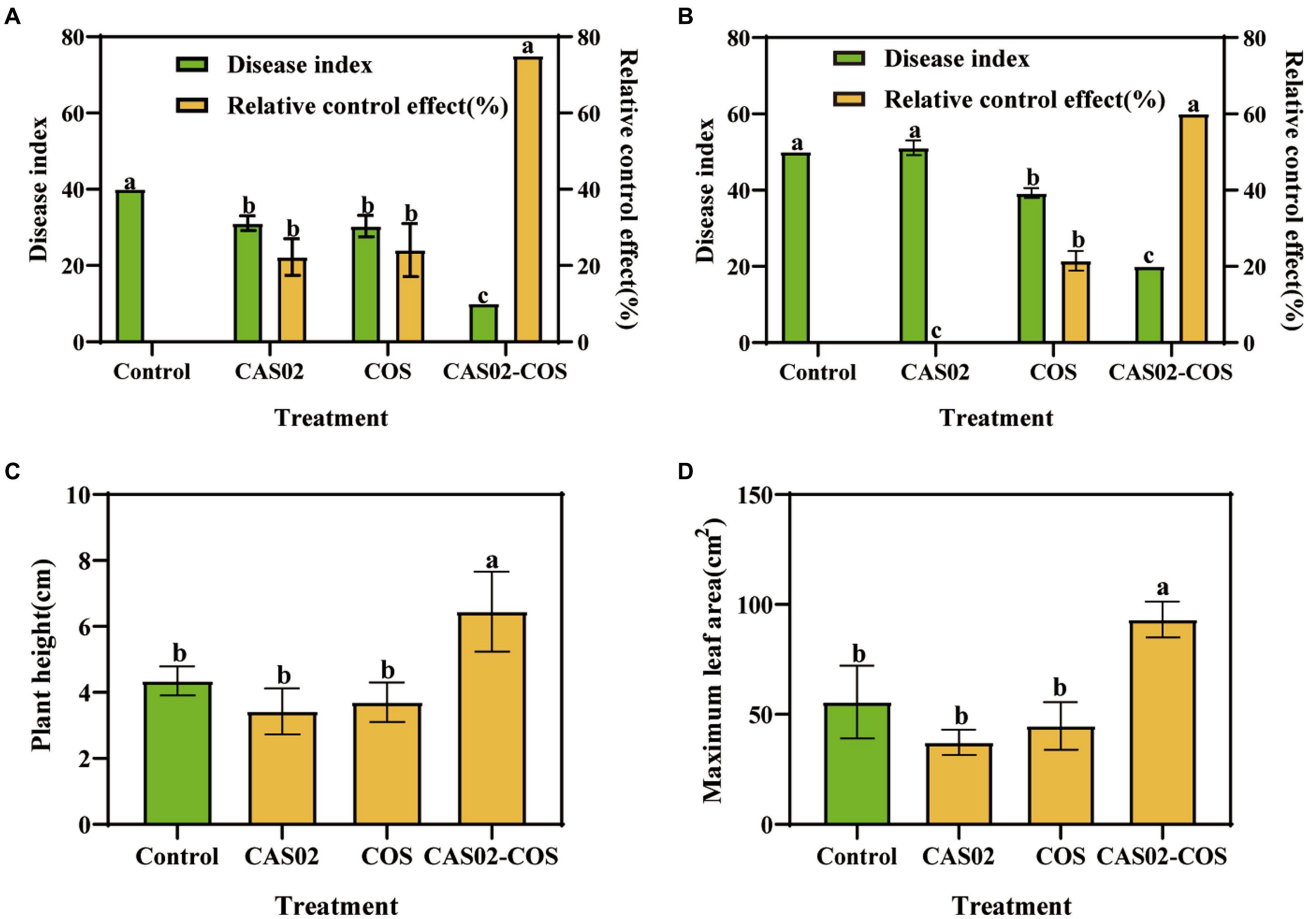
The effect of combined application of biocontrol bacteria CAS02 and COS on the prevention and control of tobacco black shank and on tobacco plant growth. Tobacco black shank disease index and relative control effect at day 10 **(A)** and 25 **(B)**. **(C)** Plant height. **(D)** Maximum leaf area. Control: No CAS02 suspension or COS added; CAS02: 25 mL CAS02 suspension (OD_600_ value of 0.4) was added; COS: 36 mg COS was added; CAS02-COS: 25 mL CAS02 suspension (OD_600_ value of 0.4) and 36 mg COS were added. Data are means ± SE (*n* = 3). Different letters on the bars indicate significant differences (*p* < 0.05).

Disease indices and relative control effect were investigated using pot experiments. At the early stage (the day 10 after *P. nicotianae* inoculation), application CAS02 or COS alone significantly decreased the disease index relative to control (Figure 4A). However, with the increase of time, this decrease in disease index gradually declined, especially for application CAS alone, which was not significant different with the control treatment on the day 25 (Figure 4B). In contrast, co-application of CAS02 and COS maintained the lowest disease index, whose relative control effect was 75% and 60% at day 10 and 25, respectively, significantly higher than application CAS02 or COS alone (Figures 4A,B). Furthermore, compared with CK, application CAS02 or COS alone did not significantly affect the agronomic traits of the tobacco plants; on the contrary, synergistic application of CAS02 and COS significantly increased the plant height and maximum leaf area of tobacco seedlings by 48.28% and 67.43%, respectively (Figures 4C,D).

### 3.5 Effects of co-application of CAS02 and COS on microbial community in rhizosphere soil

**Figure 5:**
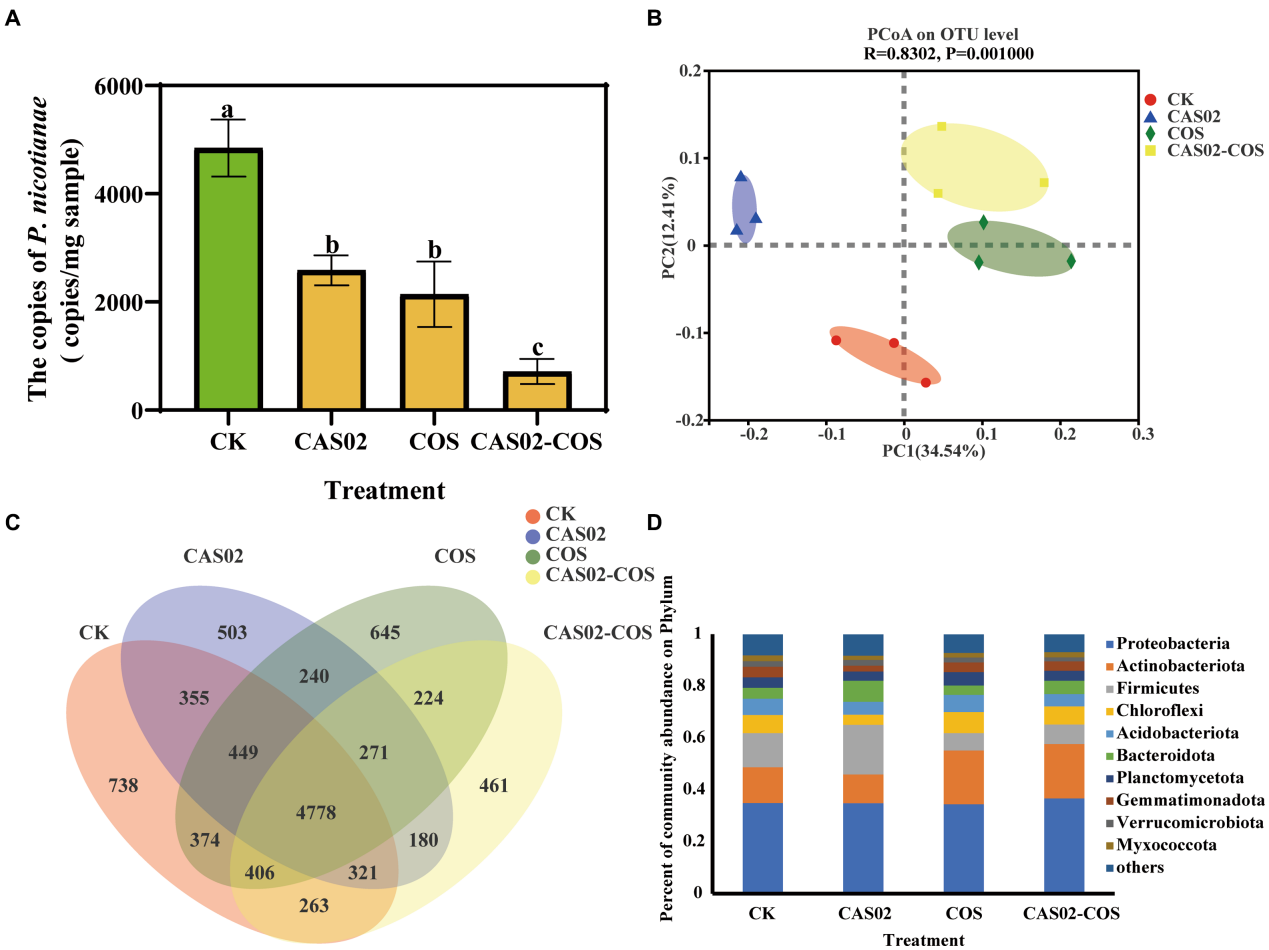
Profiles of microbial community in the rhizosphere soil in different treatments. **(A)** The density of *P. nicotianae* in the rhizosphere soil in different treatments. **(B)** Principal component analysis (PCoA) of bacterial community composition in rhizosphere soils of different treatments at OTU level. **(C)** Venn diagram showing the number of specific and shared OTUs in rhizosphere soils among different treatments; **(D)** Relative abundance of bacteria at the phylum level (top 10) in rhizosphere soils of different treatments. Control: No CAS02 suspension or COS added; CAS02: 25 mL CAS02 suspension (OD_600_ value of 0.4) was added; COS: 36 mg COS was added; CAS02-COS: 25 mL CAS02 suspension (OD_600_ value of 0.4) and 36 mg COS were added. Data are means ± SE (*n* = 3). Different letters on the bars indicate significant differences (*p* < 0.05).

As shown in Figure 5A, synergistic application of CAS02 and COS but not CAS02 or COS alone significantly reduced the density of *P. nicotianae* in the rhizosphere soil, indicating that the synergistic application of CAS02 and COS can significantly improve the relative control effect of tobacco black shank control.

The effect of co-application of CAS02 and COS on rhizosphere soil bacterial richness and diversity was investigated based on Chao 1 and Shannon diversity index. As shown in Supplementary Figure S3, the Chao 1 and Shannon diversity index showed no significant differences among the four treatments, indicating that application of CAS02 and COS individually or combinedly had no significant effects on bacterial richness and diversity. According to the result of PCoA (Figure 5B), the first two principal components explained 46.95% of the variation of the total rhizosphere soil bacterial communities. The samples from different treatments were clearly separated with each other, evidencing the significant shift of bacterial community composition with individual or co-application of COS and CAS02. Venn diagrams showed that there were 738, 503, 645 and 461 unique OTUs observed in control, CAS02, COS, and CAS02-COS treatment, respectively (Figure 5C). Furthermore, as shown in Figure 5D, at the phylum level, Proteobacteria, Actinobacteriota and Firmicutes were dominant in rhizosphere soil bacterial community, of which, Actinobacteriota and Firmicutes showed significantly varying relative abundance among different treatments (Figure 5D).

**Figure 6:**
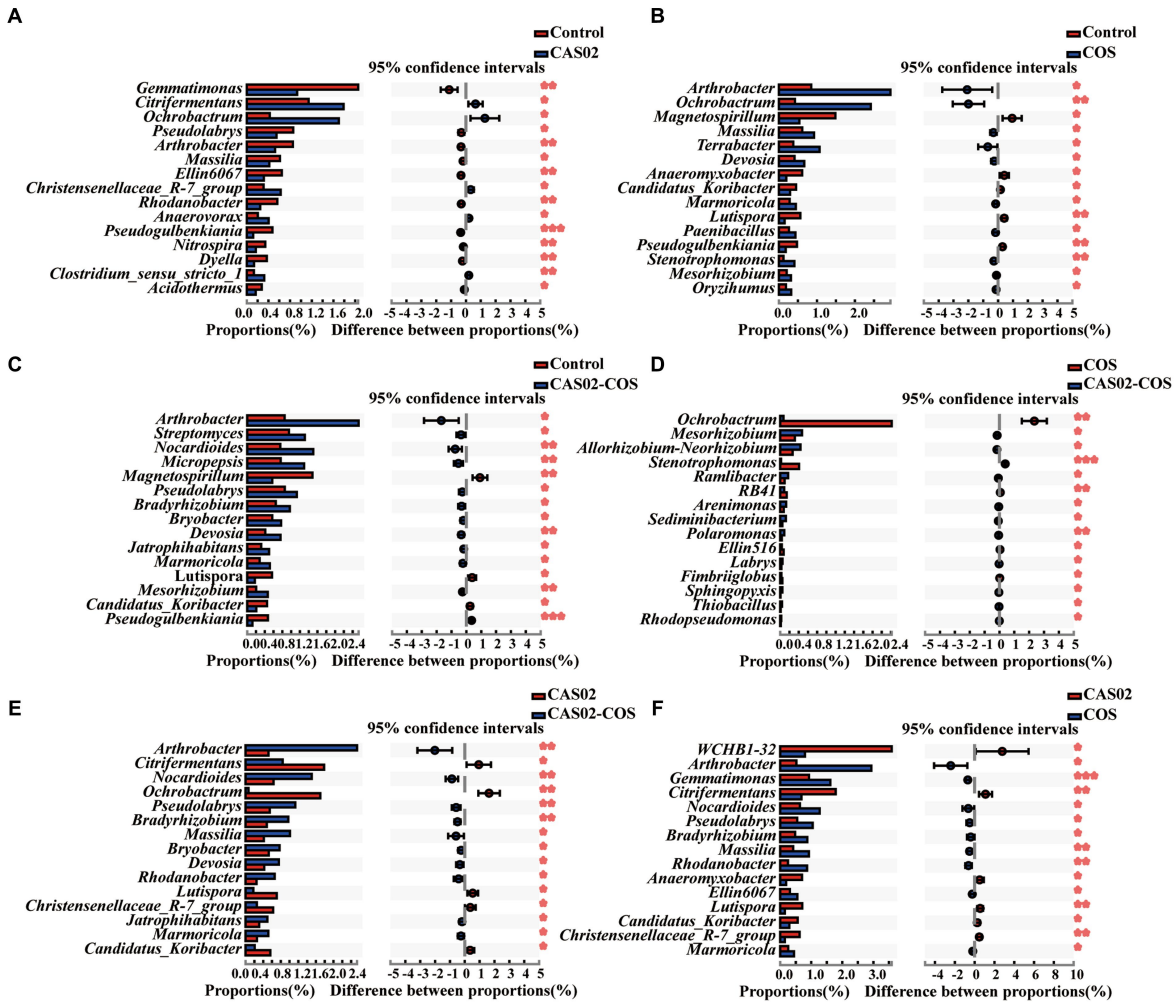
Two group comparisons showing the difference in the relative abundance of bacterial genera (top 15) in rhizosphere soils among different treatments. The distribution of the relative abundance of bacterial taxa between control and individual or co-application of CAS02 and COS **(A–C)** and between individual and co-application of CAS02 and COS **(D–F)**. Control: No CAS02 suspension or COS added; CAS02: 25 mL CAS02 suspension (OD_600_ value of 0.4) was added; COS: 36 mg COS was added; CAS02-COS: 25 mL CAS02 suspension (OD_600_ value of 0.4) and 36 mg COS were added.

Spearman’s correlation analysis was used to investigate the correlation between the disease indices and the relative abundance of microbial taxa at genus level. The results showed that the relative abundance of *Nocardioides, Arthrobacter*, *Devosia* and *Bradyrhizobium* was significantly negatively correlated with the disease index (Supplementary Table S5). Moreover, the relative abundance of *Nocardioides*, *Devosia*, and *Bradyrhizobium* was significantly higher in the CAS02-COS treatment than in the other treatments (Supplementary Figure S4). To further investigate the response of bacterial taxa to the individual or combined application of CAS02 and COS, two group comparisons were conducted at genus level using Student’s *t*-test (Figure 6). Compared with control, there are 5 and 10 bacterial genera, whose relative abundance was significantly increased with CAS02 and COS application alone, respectively (Figures 6A,B). However, more bacterial taxa (11 genera) with significant increased relative abundance were obtained after co-application of CAS02 and COS, including *Streptomyces*, *Pseudolabrys* and *Mesorhizobium*, compared to the control (Figure 6C). There were 10 (e.g., *Ramlibacter*, *Sphingopyxis*, and *Rhodopseudomonas*) and 10 (e.g., *Rhodanobacter*) bacterial genera significantly enriched after co-application of CAS02 and COS compared with application COS or CAS02 alone, respectively (Figures 6D,E).

### 3.6 Effects of co-application of CAS02 and COS on the relative expression of defense signal marker genes in tobacco plants

**Figure 7:**
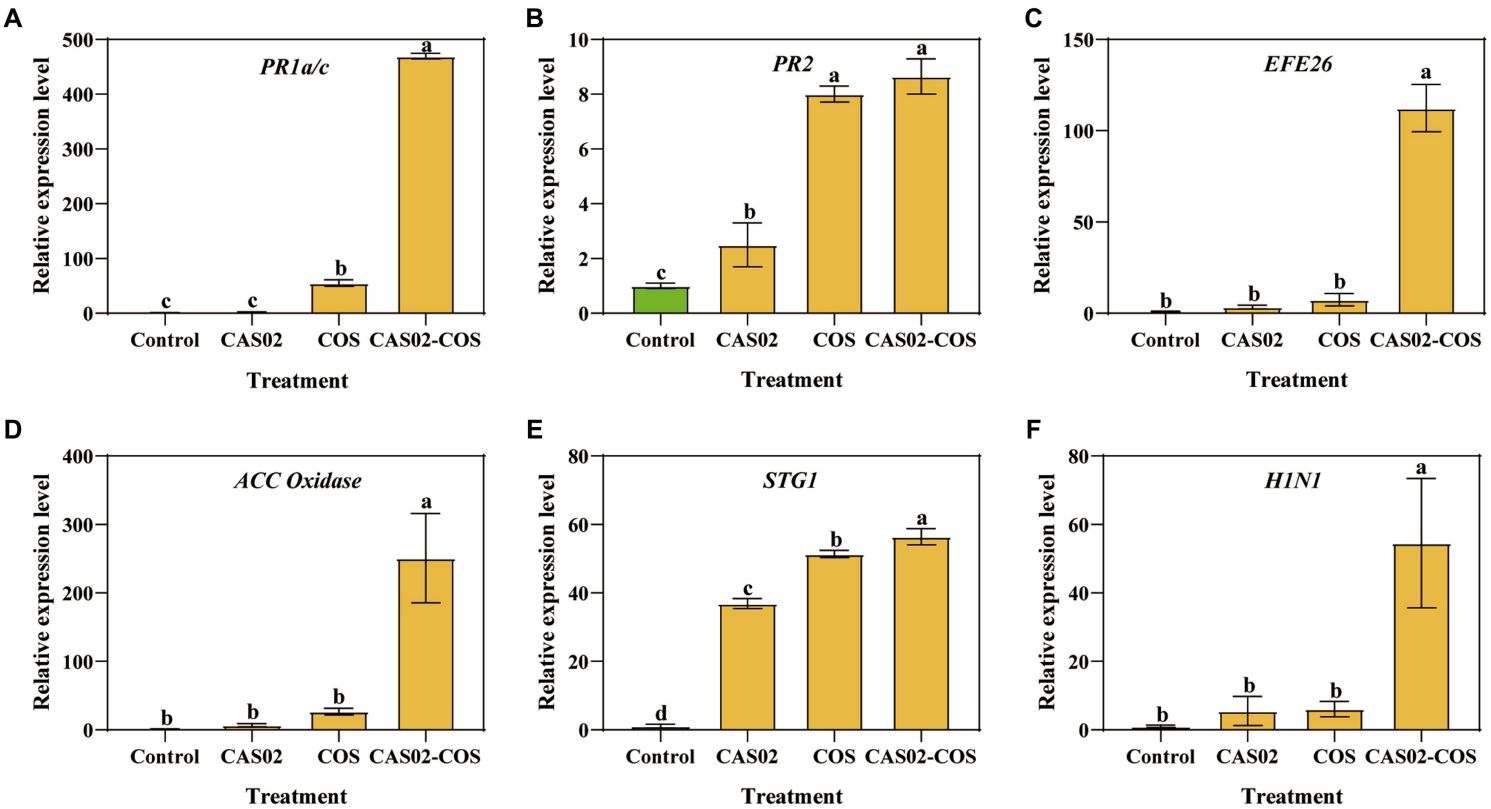
Relative expression levels of defense signaling marker genes. **(A)** PR1a/c. **(B)** *PR2*. **(C)** *EFE26*. **(D)** *ACC oxidase*. **(E)** *SGT1*. **(F)** *H1N1*. Control: No CAS02 suspension or COS added; CAS02: 25 mL CAS02 suspension (OD_600_ value of 0.4) was added; COS: 36 mg COS was added; CAS02-COS: 25 mL CAS02 suspension (OD_600_ value of 0.4) and 36 mg COS were added. Data are means ± SE (*n* = 3). Different letters on the bars indicate significant differences (*p* < 0.05).

To evaluate the activation of defense signaling in tobacco plants by co-application of CAS02 and COS, the relative expression of defense signaling marker genes associated with salicylic acid (*SA*), ethylene (*ET*), and hypersensitive response (*HR*) signaling in tobacco leaves was analyzed (Jiao et al., 2020; Figure 7). Compared with the control, the relative expression levels of SA signaling marker genes *PR1a/c* and *PR2*, *ET* signaling marker genes *EFE26* and *ACC oxidase*, and *HR* signaling marker genes *SGT1* and *H1N1* were increased by 1.83–469.49 folds, 2.50–8.64 folds, 3.46–112.35 folds, 6.79–250.96 folds, 36.89–56.41 folds and 5.53–54.54 folds after individual or co-application of CAS02 and COS. Furthermore, the relative expression levels of these genes were significantly higher after co-application of CAS02 and COS than application CAS02 or COS alone. This result suggests that co-application CAS02 and COS enhanced systemic resistance of tobacco against tobacco black shank at the molecular level through *SA*, *ET*, and *HR* pathways.

## 4 Discussion

Biocontrol bacteria have been widely reported as a new approach to control plant diseases (Kumar et al., 2000; Sathiyabama and Balasubramanian, 2018; Ma et al., 2020). However, their biocontrol ability is unstable and cannot be widely applied in practical productions (Ma et al., 2015; Moreno-Velandia et al., 2021). Improving the biocontrol effect of biocontrol bacteria is the focus of this study. In previous studies, chitosan has been widely studied because of its broad-spectrum antibacterial ability (Sánchez et al., 2017; Yin et al., 2020). In this study, biocontrol bacteria CAS02 and the marine oligosaccharide COS, which have excellent ability to against *P. nicotianae*, were screened. We found that specific concentrations of COS in combination with CAS02 could cause more damage to *P. nicotianae* mycelia than COS or CAS02 alone. It may be because CAS02 and COS played a synergistic role in enhancing the biocontrol effect on *P. nicotianae* (Lin et al., 2020). A previous study also showed that the combination of the 2.0% (w/v) exogenous trehalose and the *Hansenlaspora uvarum* strain Y3 significantly disrupted the structure of *Aspergillus tubingensis* and *Penicillium commune*, thereby inhibiting the growth of pathogenic bacteria (Apaliya et al., 2018).

The results of biocontrol pot trials showed that application CAS02 or COS alone had certain effects in suppressing tobacco black shank early in the disease. However, the suppression effect tended to decrease with time. This phenomenon may be ascribed to the susceptibility of biocontrol bacteria to environmental factors and the variation in its colonization ability, which made the decline of the biological control ability of CAS02 more seriously (Fira et al., 2018). The synergistic application of CAS02 and COS compensated well for this drawback, as CAS02-COS maintained the highest relative control effect during disease onset and the suppression effect was longer-lasting. The qPCR results also supported this conclusion, as co-application of CAS02 and COS resulted in the lowest density of *P. nicotianae* in the rhizosphere soil compared to application COS or CAS02 alone. Similarly, a previous study also demonstrated that synergistic application of trehalose with the biocontrol bacteria *R. glutens* and *C. laurentii* significantly improved the biological control of apple blue mold (Apaliya et al., 2018). Furthermore, although strain Cas02 was reported to produce Indole acetic acid (IAA), siderophore and ammonia that potentially promote the plant growth (Yuan et al., 2021; Chu et al., 2022), application of Cas02 alone showed little impact on the plant growth in current study, while co-application of CAS02 and COS significantly increased the plant height and maximum leaf area. This phenomenon may be ascribed to that the strain Cas02 fails to compete with indigenous soil microorganisms, and some of which may eventually die during the adaptation to the environment (Ahmed et al., 2023). While co-application of CAS02 and COS may enhance the compete ability of CAS02 (Rush et al., 2022), eventually result in a synergistic effect on plant growth promotion.

The changes in rhizosphere soil microbial communities are closely related to the occurrence of plant diseases (Berendsen et al., 2018; Zheng et al., 2021). After co-application of CAS02 and COS, the rhizosphere soil microbial communities changed, which reduced black shank caused by *P. nicotianae* and enhanced the suppressive effect on root diseases. We found that co-application of CAS02 and COS significantly increased the relative abundance of *Nocardioides*, *Devosia*, and *Bradyrhizobium*, three bacterial genera, significantly and negatively correlated with disease indices. Previous studies have shown that *Nocardioides* can effectively suppress tomato wilt fungus (Zhao F. et al., 2019; Fu et al., 2021). *Devosia* can be potentially valuable for controlling plant diseases by acting as a root regulator to promote healthy plant growth (Mannaa et al., 2020). *Bradyrhizobium* has a potential role in inhibiting tobacco black shank (Liu et al., 2020). In addition, *Nocardioides*, *Devosia* and *Bradyrhizobium* are all associated with the soil nitrogen cycling, which can effectively improve the soil microhabitat, promote plant growth and development (Verginer et al., 2010; Qiu et al., 2018; Xu et al., 2018; Yoneyama et al., 2019; Pang et al., 2022).

In current study, co-application of CAS02 and COS also significantly enhanced the relative abundance of some other bacterial genera with potential disease suppressive activity. Both *Pseudolabrys* and *Mesorhizobium* can control plant diseases by suppressing pathogenic bacteria and also have positive effects on promoting plant growth and development (Cipriano et al., 2016; Das, 2017). *Streptomyces* can act as a biocontrol agent by producing other bioactive metabolites and antibiotics (Bai et al., 2015; Barka et al., 2016). In addition, the effectiveness of *Rhodanobacter*, *Ramlibacter*, *Sphingopyxis*, and *Rhodopseudomonas* in suppressing plant diseases has been reported. *Rhodanobacter* can effectively suppress several pathogenic bacteria (Zhang et al., 2020). Similarly, *Sphingopyxis* can be used as a biocontrol agent in combination with other bacteria to control plant diseases (Fujiwara et al., 2016). *Rhodopseudomonas* and *Ramlibacter* play essential role in plant disease control via enhancing the immune response and promoting plant growth (Zhang et al., 2020). In summary, these results suggest that CAS02 and COS applied synergistically can recruit beneficial and antagonistic bacterial communities, thus indirectly suppressing the occurrence of tobacco black shank.

The plant defense system, generally includes physical and chemical barriers that preexists, and inducible defense responses (Liu et al., 2013). Induced systemic resistance (ISR) could induce the entire plant body against a broad range of pathogens (Pieterse et al., 2014). In tobacco plants, local and systemic defense responses could be mediated by the *SA*, *ET* and *HR* signaling pathways (Tahir et al., 2017; Zhao L. et al., 2019; Li et al., 2020). Previous studies have shown that bio-stimulatory factors can enhance plant resistance to pathogens by activating the expression of defense signaling marker genes (Tahir et al., 2017; Jiao et al., 2020). In current study, the synergistic application of CAS02 and COS substantially increased the relative expression levels of *SA* signaling marker genes, *ET* signaling marker genes, and *HR* signaling marker genes compared to CAS02 and COS alone (Figure 7). Moreover, *SA*, *ET*, and *HR* pathways played an essential role in enhancing disease resistance in tobacco plants (Shang et al., 2021). This suggests that CAS02 and COS also synergistically induce the tobacco plant to improve resistance to tobacco black shank, although this resistance should be further detected by inoculation phytopathogen on the leaf and the expression of proteins should also be confirmed by biochemistry in future studies.

## 5 Conclusion

In this study, we found that the screened *Bacillus amyloliquefaciens* CAS02 and chitooligosaccharide had strong *in vitro* resistance against *P. nicotianae*. The synergistic effect of CAS02 and COS significantly enhanced the inhibitory activity against *P. nicotianae* through multiple mechanisms. The synergistic application of CAS02 and COS significantly increased the relative abundance of beneficial microbial taxa such as *Nocardioides*, *Devosia*, and *Bradyrhizobium* in the rhizosphere soil. Furthermore, the synergistic effect of the two substantially activated the relative expression level of defense signaling marker genes of *SA*, *ET*, and *HR* pathways. The combined effect of CAS02 and COS application significantly improved the relative control effect against tobacco black shank. However, the underlying mechanism should be further explored. Future work will focus on the effect and underlying mechanism of co-application of CAS02 and COS on the production of antifungal active compound and root colonization of CAS02. This study provide evidence for synergistic efficacy of biocontrol bacteria and chitooligosaccharide as a promising strategy for controlling tobacco black shank.

## Data availability statement

The datasets presented in this study can be found in online repositories. The names of the repository/repositories and accession number(s) can be found here: https://bigd.big.ac.cn/gsa, CRA013338.

## Author contributions

XZe: Methodology, Visualization, Writing – original draft. XZh: Visualization, Formal analysis, Methodology, Writing – original draft. BP: Methodology, Writing – original draft. BX: Methodology, Writing – original draft. YY: Formal analysis, Methodology, Writing – original draft. HY: Visualization, Writing – review & editing. XY: Writing – original draft, Conceptualization, Project administration, Supervision. JW: Writing – review & editing. YL: Funding acquisition, Writing – original draft.
