# Supplementary material for: Chitooligosaccharide enhanced the efficacy of Bacillus amyloliquefaciens CAS02 for the control of tobacco black shank
Source: Front Microbiol. 2023 Nov 24;14:1296916. doi: 10.3389/fmicb.2023.1296916 (PMC10704449; doi:10.3389/fmicb.2023.1296916)
Supplement: Supplementary file 1 [file Data_Sheet_1.DOCX]

Supplementary Materials for

**Chitooligosaccharide enhanced the efficacy of *Bacillus amyloliquefaciens* CAS02 for the control of tobacco black shank**

**Xiangnan Zeng^1†^, Xin Zhang^2†^, Bo Peng^1^, Bingyue Xie^1^, Yuan Yuan^2^, Hui Yao^2*^, Xiangwei You^2^, Jianyu Wei^1*^, Yiqiang Li^2^**

^1^China Tobacco Guangxi Industrial Co., Ltd., Nanning 530001 China

^2^Marine Agriculture Research Center, Tobacco Research Institute, Chinese Academy of Agricultural Sciences, Qingdao 266101, China

†These authors contributed equally to this work and share first authorship

†These authors contributed equally to this work and share first authorship

^*^Corresponding authors:

Hui Yao; yaohui@caas.cn

Jianyu Wei; jtx_wjy@163.com

**The supplementary figures include:**

**FIG S1** (A) The relative inhibition rate of different concentrations of COS against P. nicotianae. (B) Toxicity regression equation.

**FIG S2** Effect of COS addition on growth curve and morphology of biocontrol bacteria CAS02. (A) Growth curve. Morphology of biocontrol bacteria CAS02 on medium without COS (B) and added with COS at 188 μg/mL (C).

**FIG S3** The effect of combined application of biocontrol bacteria CAS02 and COS on the rhizosphere soil bacterial richness and diversity. Bars with shared uppercase and lowercase letters indicate no significant differences in Chao 1 and Shannon diversity index among treatments.

**FIG S4** Relative abundance of specific rhizosphere microorganisms that are negatively correlated with disease index in the rhizosphere soil in different treatments. Control: No CAS02 suspension or COS added; CAS02: 25 mL of CAS02 suspension (OD_600_ value of 0.4) was added; COS: 36 mg COS was added; CAS02-COS: 36 mg COS and 25 mL CAS02 suspension (OD_600_ value of 0.4) were added.

**The additional tables include:**

**Table S1** The source of eight biocontrol bacteria for testing in this study.

**Table S2** Primers used in this study.

**Table S3** The relative inhibition rate of different biocontrol bacteria against *P. nicotianae*.

**Table S4** The relative inhibition rate of different marine polysaccharides/oligosaccharides against *P. nicotianae*.

**Table S5** Correlation analysis between specific rhizosphere microorganisms and disease index.

**
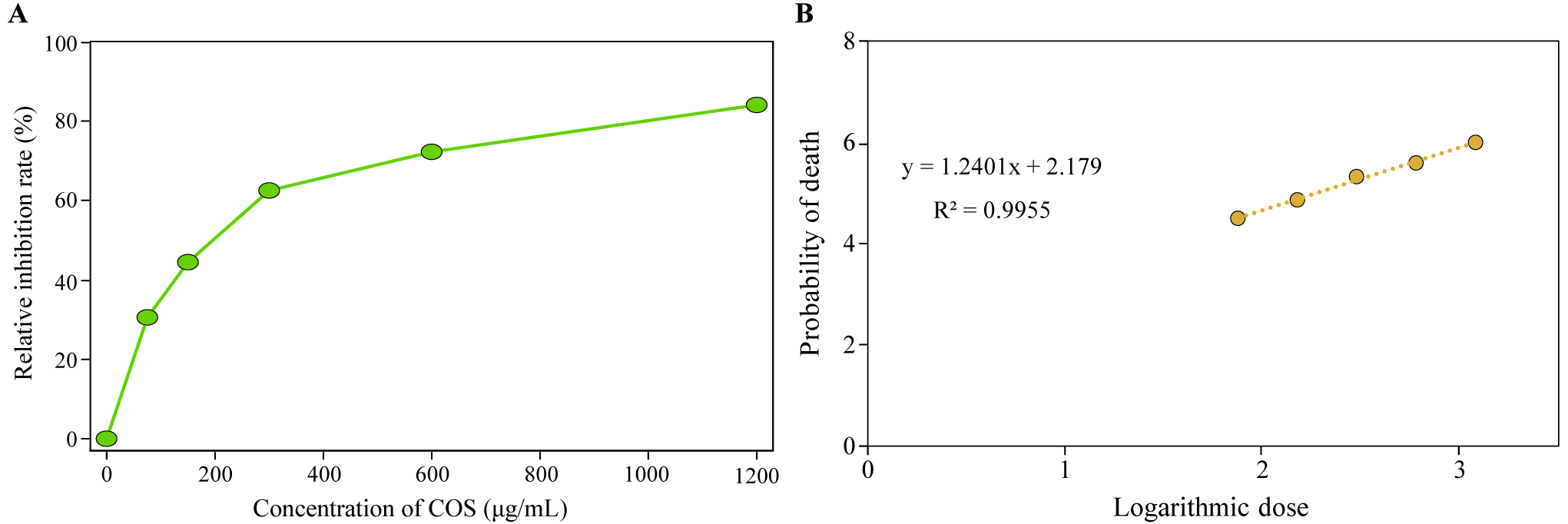
**

**FIG S1** (A) The relative inhibition rate of different concentrations of COS against *P. nicotianae*. (B) Toxicity regression equation.


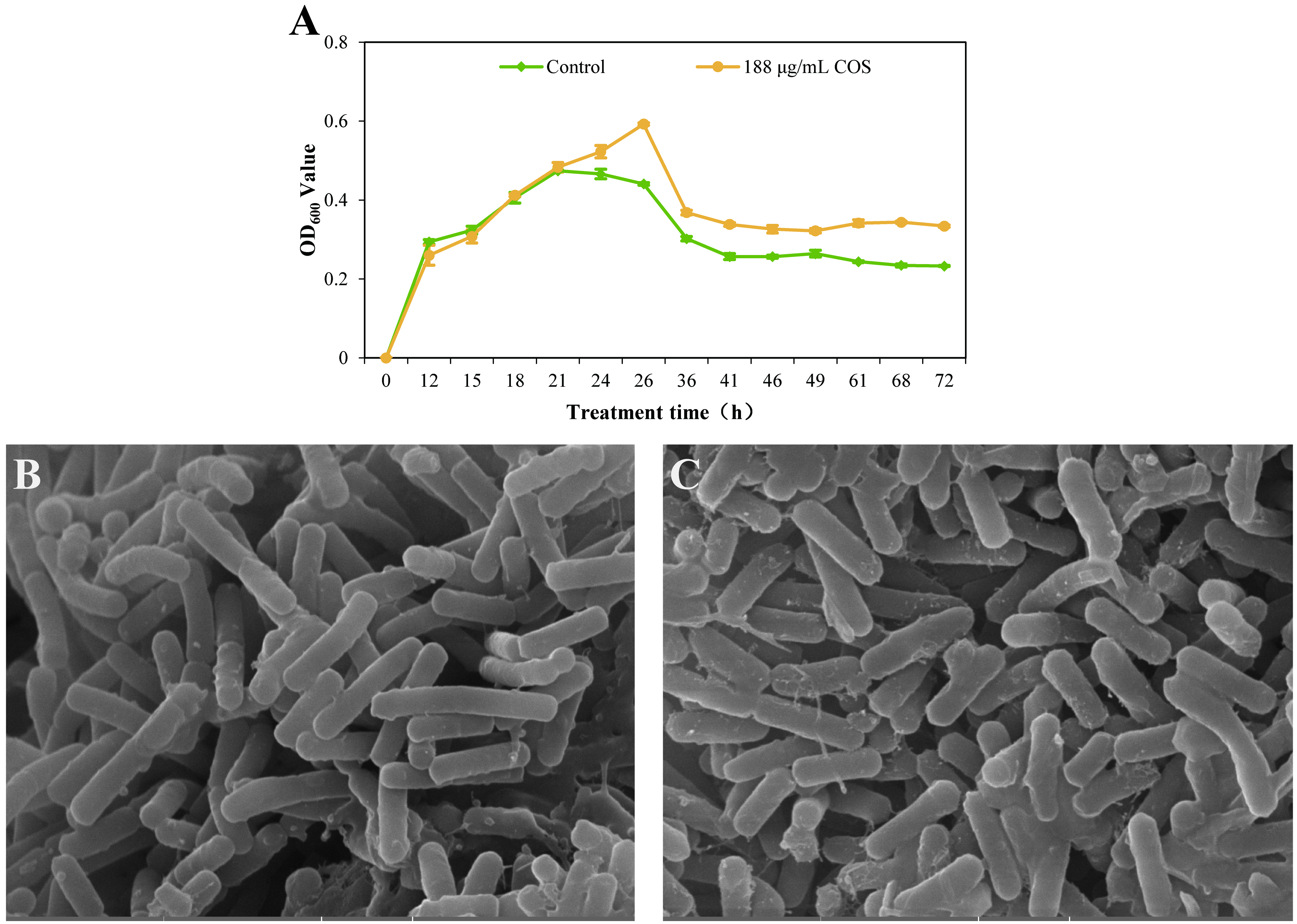


**FIG S2** Effect of COS addition on growth curve and morphology of biocontrol bacteria CAS02. (A) Growth curve. Morphology of biocontrol bacteria CAS02 on medium without COS (B) and added with COS at 188 μg/mL (C).


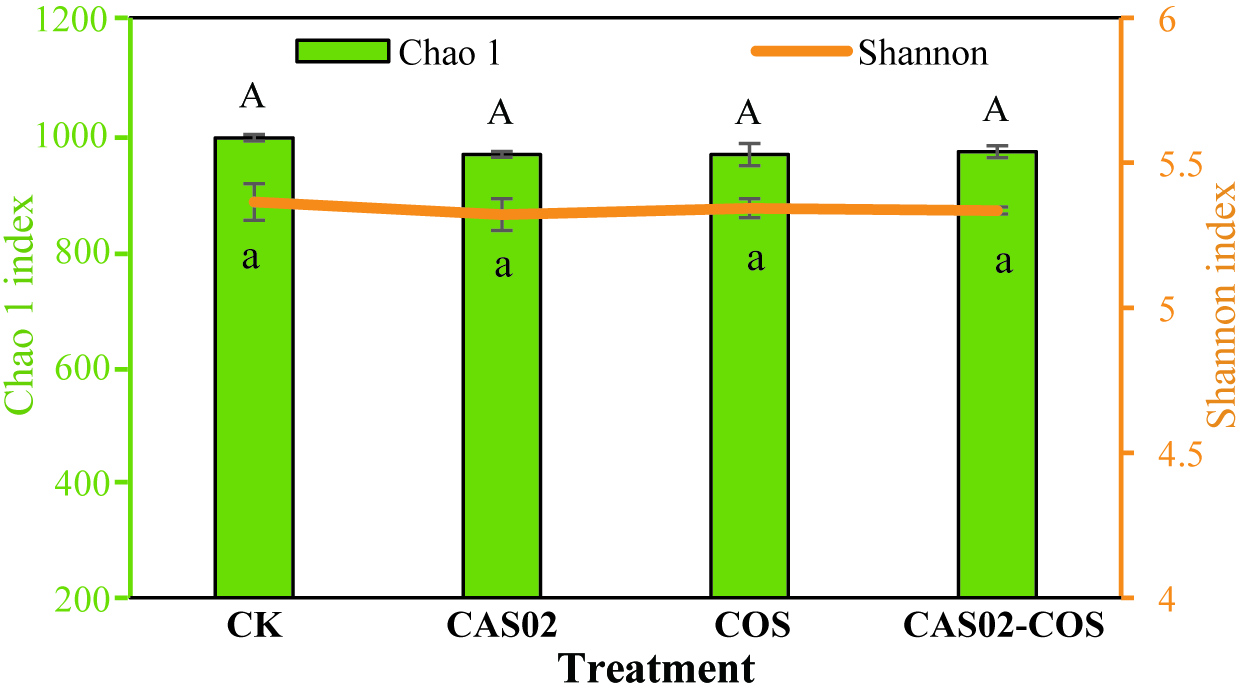


**FIG S3** The effect of combined application of biocontrol bacteria CAS02 and COS

on the rhizosphere soil bacterial richness and diversity. Bars with shared uppercase

and lowercase letters indicate no significant differences in Chao 1 and Shannon diversity index among treatments.


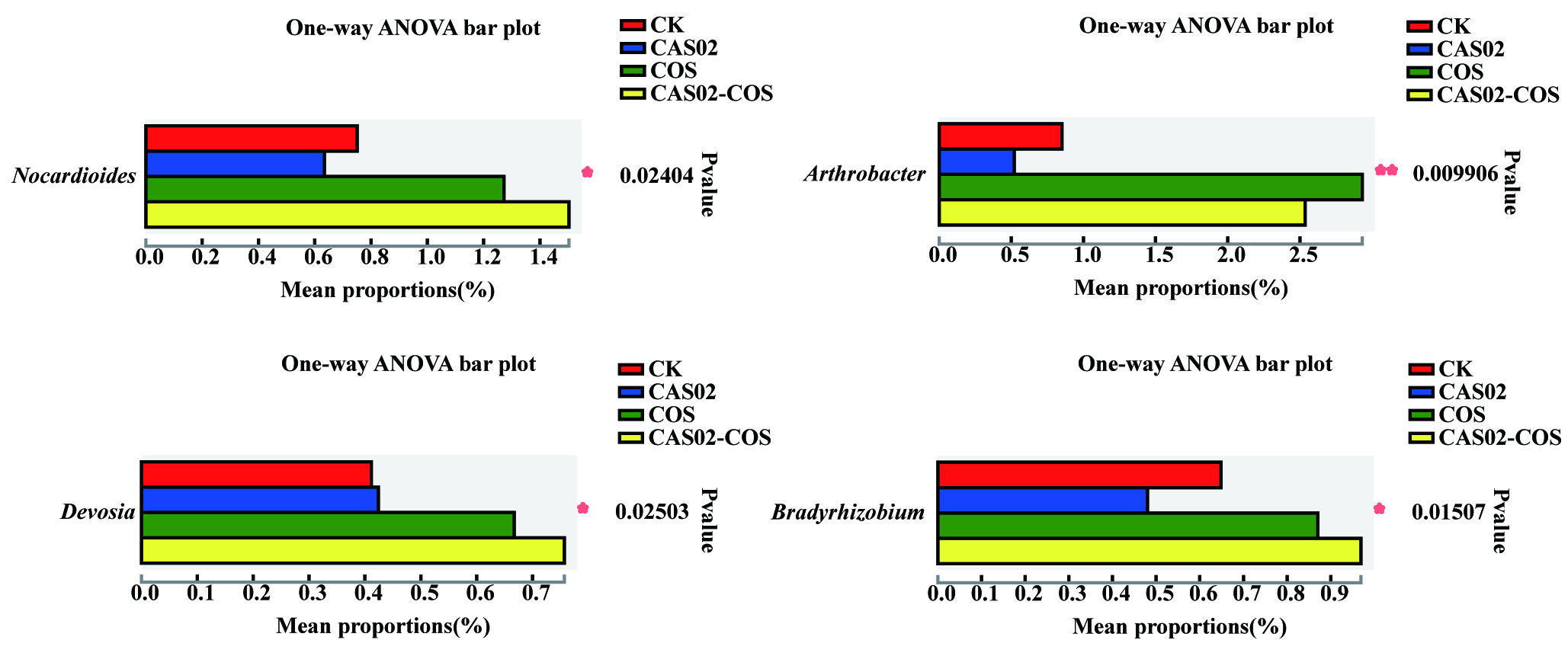
**FIG S4** Relative abundance of specific rhizosphere microorganisms that are negatively correlated with disease index in the rhizosphere soil in different treatments. Control: No CAS02 suspension or COS added; CAS02: 25 mL CAS02 suspension (OD_600_ value of 0.4) was added; COS: 36 mg COS was added; CAS02-COS: 36 mg COS and 25 mL CAS02 suspension (OD_600_ value of 0.4) were added.

**Table S1** The source of eight biocontrol bacteria for testing in this study.

| Strains | Isolated sources |
| --- | --- |
| *Bacillus amyloliquefaciens* CAS02 | Rhizosphere soils of healthy tobacco plants |
| *Bacillus aureus* 3T33 | Soil |
| *Bacillus megaterium* BB22 | Soil |
| *Bacillus adenii* BBS23 | Soil |
| *Bacillus natto* NATT0 | Natto |
| *Bacillus alpine* 7 | Hot spring |
| *Bacillus amyloliquefaciens* SB1 | Soybean organic fertilizer |
| *Bacillus amyloliquefaciens* SB2 | Soybean organic fertilizer |

**Table S2** Primers used in this study.

| Gene | Forward primer | Reverse primer |
| --- | --- | --- |
| *PR1 a/c* | AACCTTTGACCTGGGACGAC | GCACATCCAACACGAACCGA |
| *PR2* | TGATGCCCTTTTGGATTCTATG | AGTTCCTGCCCCGCTTT |
| *EFE26* | CGGACGCTGGTGGCATAAT | CAACAAGAGCTGGTGCTGGATA |
| *ACC Oxidase* | GACAAAGGGACATTACAAGAAGT | GAGAAGGATTATGCCACCAG |
| *H1N1* | CGACCTAACAAAGTCAAGTTCTACG | CTCTATCTCCCAATAAAACCAAGC |
| *STG1* | TGCTGCTGTAACAAGATGGATGC | GAGATGGGGACAAAGGGGATT |

**Table S3** The relative inhibition rate of different biocontrol bacteria against *P. nicotianae*.

| Treatment | Relative inhibition rate (%) |
| --- | --- |
| Control | - |
| CAS02 | 72.79 ± 0.00a |
| 3T33 | 0.74 ± 0.00f |
| BB22 | 0.00 ± 0.00g |
| BBS23 | 0.00 ± 0.00g |
| NATT0 | 65.56 ± 0.32c |
| 7.00 | 61.89 ± 0.12e |
| SB-1 | 66.91 ± 0.00b |
| SB-2 | 64.46 ± 0.25d |

CAS02, *Bacillus amyloliquefaciens* CAS02; 3T33, *Bacillus aureus* 3T33; BB22, *Bacillus megaterium* BB22; BBS23, *Bacillus adenii* BBS23, NATT0; *Bacillus natto* NATT0; 7, *Bacillus alpine* 7; SB1, *Bacillus amyloliquefaciens* SB1; SB2, *Bacillus amyloliquefaciens* SB2. Different letters indicate significant differences (*P* < 0.05).

**Table S4** The relative inhibition rate of different marine polysaccharides/oligosaccharides against *P. nicotianae*.

| Treatment | Relative inhibition rate (%) |
| --- | --- |
| Control | - |
| COS | 100.00 ± 00.00a |
| EP | 34.71 ± 0.34c |
| FOS | 40.98 ± 0.20b |
| AOS | 27.84 ± 0.85e |
| KOS | 29.61 ± 0.20d |

COS, chitooligosaccharide; AOS, alginate oligosaccharides; CAOS carrageenan oligosaccharide; EP, enteromorpha polysaccharide; FOS, fucoidan oligosaccharide. Different letters indicate significant differences (*P* < 0.05).

**Table S5** Correlation analysis between specific rhizosphere microorganisms and disease index.

| Bacteria taxa |  | r | *P* value |
| --- | --- | --- | --- |
| *Nocardioides* |  | -0.87477 | 0.0002** |
| *Arthrobacter* |  | -0.77595 | 0.00301** |
| *Devosia* |  | -0.71498 | 0.00896** |
| *Bradyrhizobium* |  | -0.66248 | 0.0189* |
